# Hepatitis C as a metabolic disease

**DOI:** 10.1186/1742-4690-9-S1-I17

**Published:** 2012-05-25

**Authors:** Patrice Andre

**Affiliations:** 1 Croix-Rousse Hospital, Lyon, France

## 

Before liver cirrhosis and hepatocellula carcinoma can develop, the early and long lasting features of hepatitis C are host metabolism modifications with a specific and so far unique metabolic syndrome that may associate insulin resistance, liver steatosis and hypo-betalipoproteinemia. These metabolic perturbations are directly induced by heptatitis C virus (HCV) replication and regress after viral suppression. Symmetrically HCV depends on glucose and lipid metabolism for its replication.

HCV induced insulin resistance is both hepatic and peripheral. The insulin receptor pathway is impacted at several steps by viral proteins with probable functional consequences. Mechanism of peripheral insulin resistance remains obscure and the viral signals send to adipose tissue or muscles have to be identified. One major IR metabolic consequence might contribute to the mobilization of free fatty acids from periphery to the liver and to the constitution of liver steatosis. Importantly, HCV modifies with genotype-specific differences, the synthesis, degradation and secretion of lipid in a coordinated fashion to promote the accumulation of neutral lipids.

The lipid droplets (LD) that are the lipid storage organelles are mandatory platforms for the assembly of infectious viral particles. In particular localization of core protein and NS5A on LD and mobilization of the LD are essential steps that control viral infectivity. Interestingly, the efficiency of HCV to the LD disposal and use correlates to viral production and may influences the extent of hepatic accumulation of lipids.

The most striking association of HCV with lipid metabolism resides in the coincidence of the betalipoproteins and viral particles pathways with the formation of unique hybrid viral particles. Indeed HCV depends on a functional very low density lipoproteins (VLDL) assembly and secretion process to be secreted. HCV also modifies at different degree the VLDL composition forming sub viral particles, which are minimally modified VLDL that bear the viral envelop glycoproteins, or hybrid viral particles known as lipo-viral-particles (LVP), which contain all the viral and VLDL components that are highly infectious. Functions of these hybrid particles on the disease features and progression as well as therapeutic targets remain to be fully characterized.

Thus, HCV appears to have developed an original and so far unique way with major clinical consequences to modify and use the lipid metabolism to persist in the host.

